# The 18-kDa Translocator Protein (TSPO) Disrupts Mammary Epithelial Morphogenesis and Promotes Breast Cancer Cell Migration

**DOI:** 10.1371/journal.pone.0071258

**Published:** 2013-08-14

**Authors:** Xiaoting Wu, Kathleen A. Gallo

**Affiliations:** 1 Cell and Molecular Biology Program, Michigan State University, East Lansing, Michigan, United States of America; 2 Department of Physiology, Michigan State University, East Lansing, Michigan, United States of America; Baylor College of Medicine, United States of America

## Abstract

Mitochondria play important roles in cancer progression and have emerged as viable targets for cancer therapy. Increasing levels of the outer mitochondrial membrane protein, 18-kDa translocator protein (TSPO), are associated with advancing breast cancer stage. In particular, higher TSPO levels are found in estrogen receptor (ER)-negative breast tumors, compared with ER-positive tumors. In this study, we sought to define the roles of TSPO in the acquisition of breast cancer malignancy. Using a three-dimensional Matrigel culture system, we determined the impact of elevated TSPO levels on mammary epithelial morphogenesis. Our studies demonstrate that stable overexpression of TSPO in mammary epithelial MCF10A acini drives proliferation and provides partial resistance to luminal apoptosis, resulting in enlarged acinar structures with partially filled lumen that resemble early stage breast lesions leading to breast cancer. In breast cancer cell lines, TSPO silencing or TSPO overexpression significantly altered the migratory activity. In addition, we found that combination treatment with the TSPO ligands (PK 11195 or Ro5-4864) and lonidamine, a clinical phase II drug targeting mitochondria, decreased viability of ER-negative breast cancer cell lines. Taken together, these data demonstrate that increases in TSPO levels at different stages of breast cancer progression results in the acquisition of distinct properties associated with malignancy. Furthermore, targeting TSPO, particularly in combination with other mitochondria-targeting agents, may prove useful for the treatment of ER-negative breast cancer.

## Introduction

Breast cancer is the second most frequently diagnosed cancer and one of the leading causes of cancer death among U.S. women [1]. Estrogen receptor (ER)-negative breast cancers are typically more aggressive than ER-positive tumors [2], [3]. In the absence of HER2 overexpression, there are no currently available targeted therapies to treat ER-negative breast cancer. Chemotherapeutic agents can be useful in treating patients with ER-negative breast tumors but resistance and toxicity limit efficacy [1], [2], [4]. Mitochondria play central roles in regulating bioenergetics, metabolism and cell death. Dysregulation of mitochondria in cancer contributes to the acquisition of multiple malignant phenotypes, including aberrant proliferation, impaired apoptosis and enhanced invasion and metastasis [5]–[7]. Therefore, targeting mitochondria has emerged as a potential strategy for breast cancer therapy [5], [7].

Translocator protein (TSPO), first known as the peripheral-type benzodiazepine receptor, is a five-transmembrane domain protein that resides primarily in the outer mitochondrial membrane [8], [9]. As a component of the mitochondrial permeability transition pore (PTP) complex, TSPO is believed to be involved in the opening of the PTP, a critical step in initiating apoptosis [10]–[12]. In addition, TSPO participates in multiple cellular activities, including cholesterol transport, steroidogenesis, cell proliferation, and cellular respiration [8]. Elevated TSPO levels are found in multiple types of cancer. Increased TSPO levels are found in both prostate and colorectal tumors compared with their surrounding non-tumoral tissues [13]–[15]. Progressive elevation of TSPO levels is associated with the degree of invasiveness of breast cancer [13], [15], [16]. For instance, higher levels of TSPO are found in ductal carcinoma *in situ* (DCIS) compared with normal breast tissue; and invasive breast tumors have higher TSPO than do DCIS. In particular, higher TSPO is found in ER-negative than in ER-positive breast tumors and cell lines [13], [16], [17]. Overexpression of TSPO increases proliferation of ER-positive, luminal MCF7 cells, whereas silencing of TSPO leads to a decrease of proliferation of ER-negative, claudin-low MDA-MB-231 cells [18].

Synthetic TSPO ligands have been reported to inhibit proliferation and induce apoptosis in multiple cancer cell lines, including MCF7 cells [19]. Both the isoquinoline PK 11195 and the benzodiazepine Ro5-4864 facilitate apoptosis induced by certain chemotherapeutic agents [20]–[22]. For instance, PK 11195 sensitizes human hepatocellular carcinoma cells to apoptosis induction by paclitaxel, docetaxel, and doxorubicin [21].

The functional impact of increased TSPO levels on mammary morphogenesis and early stage breast cancer has not been investigated. The morphogenesis of mammary epithelial cells in 3D Matrigel culture shares many features with mammary gland development *in vivo* and hence has been used to investigate the impact of oncogenes on breast cancer development [23], [24]. In 3D Matrigel, a single immortalized, non-transformed mammary epithelial MCF10A cell undergoes a well-defined morphogenic program to form a growth-arrested, well-polarized, hollow acinus that resembles the acinar structure of mammary lobules [23]–[25]. During MCF10A 3D morphogenesis, proliferation continues for about 15 days and diminishes thereafter. Apoptosis of luminal cells typically initiates between day 6 and day 8, and luminal clearance is complete by about day 20 yielding hollow acinar structures. Cessation of proliferation as well as apoptosis of luminal cells is required for lumen formation [26]. Overexpression of certain oncogenes in MCF10A, including ErbB2/HER2, leads to increased proliferation and deficient luminal apoptosis in 3D Matrigel, resulting in enlarged structures with filled lumens, resembling phenotypes found in early stages of breast cancer, such as atypical hyperplasia and DCIS [26]–[28].

To better understand the potential roles of TSPO in breast cancer development and progression, the morphogenesis of MCF10A cells stably overexpressing TSPO was evaluated in 3D Matrigel culture. MCF10A-TSPO cells develop larger acini with enhanced proliferation and impaired luminal apoptosis when compared to control MCF10A acini. We also demonstrate that increased TSPO levels promote breast cancer cell migration, suggesting that TSPO may contribute to the development of invasive breast cancer. Finally, combining TSPO ligands (PK 11195 or Ro5-4864) with the mitochondrial targeting agent, lonidamine, potentiated apoptosis in ER-negative breast cancer cell lines. These studies, taken together, provide evidence that elevation of TSPO levels is sufficient to promote multiple malignant phenotypes, including increased proliferation, resistance to apoptosis, and enhanced migration. Furthermore, TSPO ligands, in combination with other agents that target the mitochondria, might be an effective approach for treating advanced breast cancer.

## Materials and Methods

### Cell Lines, Antibodies, Chemical Compounds, and siRNAs

The human mammary epithelial cell line, MCF10A, a gift from Dr. Joan Brugge (Harvard Medical School, Boston, MA, USA), was maintained as previously described [23]. Breast cancer cell lines (MCF7, MDA-MB-231 and BT549) were obtained from ATCC (Manassas, VA, USA). MDA-MB-231, MCF7 and BT549 cells were cultured in DMEM (Gibco BRL, Paisley, PA, USA) supplemented with 10% FBS and antibiotics (Penicilin/Streptomycin, 50 μg/ml). Antibody against TSPO was obtained from Novus Biological (Littleton, CO, USA). Anti-Flag M2, HA and actin monoclonal antibodies were from Sigma-Aldrich (St Louis, MO, USA). Alexa Fluor 488-conjugated anti-mouse IgG and Alexa Fluor 680-conjugated anti-goat IgG were from Li-COR Biosciences (Lincoln, NE, USA). Horseradish peroxidase-conjugated anti-rabbit IgG was from Bio-Rad (Hercules, CA, USA). The active caspase-3 and PARP antibodies were from Cell Signaling Technologies, Inc. (Danvers, MA, USA), and the Ki67 antibody was from Abcam (Cambridge, MA, USA). PK 11195, Ro5-4864 and lonidamine were also from Sigma. TSPO siRNAs (siTSPO #1: 5′-GAGAAGGCUGUGGUUCCCC-3′ and siTSPO #2: 5′-CACUCAACUACUGCGUAUG-3′) were synthesized by Dharmacon (Lafayette, CO, USA) based upon previously published sequences [29].

### Stable Cell Populations

Stable pools of FLAG tagged TSPO-expressing and control MCF10A cells were generated after retroviral transduction with pLXSN-TSPO-FLAG or an empty pLXSN vector. The TSPO-FLAG fragment was subcloned from pLH-Z_12_I-PL^2^-TSPO-FLAG vector to the retroviral vector pLXSN to construct pLXSN-TSPO-FLAG vector. pLH-Z_12_I-PL^2^-TSPO-FLAG was constructed by PCR amplification of the TSPO coding sequence from pReceiver-TSPO-HA-HIS (a kind gift from Drs. Lookingland and Goudreau, Michigan State University, MI, USA) and subcloning into pLH-Z_12_I-PL^2^ empty vector. The coding sequence for the FLAG epitope tag was incorporated into PCR primers to generate the expression vector for TSPO with a C-terminal FLAG epitope tag. The construct was fully verified by sequencing. To generate retrovirus, pLXSN-TSPO-FLAG was transfected into the 293GPG packaging cell line (a gift from Dr. R. Mulligan, Harvard Medical School, Children's Hospital, Boston, MA, USA) [30]. The retrovirus with integrated TSPO was used to infect MCF10A to generate MCF10A-TSPO cells. The stable pools of MCF10A-TSPO cells were selected in 300 μg/ml G418 and maintained in 50 μg/ml G418. The control MCF10A cell line pool expressing the empty vector pLXSN (MCF10A-pLXSN) was generated in an analogous fashion.

### Transfection and Cell Lysis

Transfection of siRNAs (20 nm) or a universal control siRNA was performed using INTERFERin (Polyplus-transfection, New York, NY, USA) according to the manufacturer's instructions. Transfection of the TSPO expression vector or a control vector was performed using Lipofectamine 2000 (Invitrogen, Carlsbad, CA, USA) according to the manufacturer's instructions. Cells cultured on 2D plastic were lysed in Triton X-100 lysis buffer (50 mM HEPES pH 7.5, 150 mM NaCl, 1.5 mM MgCl_2_, 2 mM EGTA, 1% Triton X-100, 10% glycerol, 1 mM Na_3_VO_4_, 2 mM phenylmethylsulfonyl fluoride, and a protease inhibitor cocktail (Sigma-Aldrich, St Louis, MO, USA). Protein concentrations of cellular lysates were measured by Bradford protein assays (Biorad, Pierce, Rockford, IL, USA).

### Gel Electrophoresis and Immunoblotting Analysis

Proteins were subjected to SDS–polyacrylamide gel electrophoresis and transferred from gel to Immobilon-FL PVDF membranes (Millipore, Billerica, MA, USA). The membranes were then blocked with 5% milk or Odyssey blocking buffer (Li-COR Biosciences, Lincoln, NE, USA) and incubated with appropriate antibodies, followed by incubation with a horseradish peroxidase-conjugated or an IRDye-conjugated secondary antibody, and developed by chemiluminescence method or visualized by fluorescence using the Li-COR Odyssey infrared scanner (Li-COR Biosciences, Lincoln, NE, USA), respectively.

### Immunofluorescence

For 2D cultures, cells were seeded on coverslips for 24 h, incubated with MitoTracker Red (Sigma-Aldrich, St Louis, MO, USA) for 30 min, and fixed with 3.7% formaldehyde. After fixation, cells were permeabilized with 0.5% v/v Triton X-100 for 5 min and blocked in 4% w/v bovine serum albumin (BSA) in PBS for 30 min at room temperature. Coverslips were then incubated overnight at 4 °C with anti-FLAG antibody or anti-TSPO antibody (1∶1000 dilution) in PBS containing 2% BSA. Coverslips were washed three times for 5 min each with PBS, followed by incubation with Alexa Fluor 488-conjugated anti-mouse IgG (1∶200 dilution) or Alexa Fluor 680-conjugated anti-goat IgG (1∶500 dilution) for 60 min at room temperature. After washing three times with PBS, the cells were stained with 4′-6-diamidino-2-phenylindole (DAPI, 0.5 µg/ml) for 15 min and mounted. Images were taken using an Olympus FV1000 confocal laser scanning microscope.

### 3D Morphogenesis Assay

A single cell suspension of 3000 cells was seeded per well on solidified Matrigel (BD Biosciences, San Jose, CA, USA) in overlay medium [23], [30] (DMEM/F12 supplemented with 2% horse serum; 1.5 ng/ml EGF (Peprotech, Rocky Hill, NJ, USA); 10 μg/ml insulin; 100 μg/ml hydrocortisone; 1 ng/ml cholera toxin; 50 U/ml streptomycin/penicillin and 3% Matrigel). Cultures were replenished with fresh medium every four days [23], [30]. Phase contrast images were acquired with QCapturePro. All immunofluorescence procedures were performed as previously described [23] for antibodies against Ki-67 and cleaved caspase-3. Nuclei were stained with 5 μg/ml DAPI and cells were mounted with anti-fade reagent Fluoromount-G (Southern Biotech, Birmingham, AL, USA). Fluorescence microscopy was performed using an Olympus Fluoview laser scanning microscope. Acinar structures were analyzed with ImageJ to determine size, by digitally tracing the circumference of acini and expressing the cross sectional area as pixels. Statistical analysis was performed with Student's *t*-test.

### Transwell Migration

Chemotactic transwell migration was performed using Boyden transwell chambers (8 μm pore size; Corning Costar, Cambridge, MA, USA). Cells were deprived of serum overnight, trypsinized and introduced into the upper chamber (5×10^4^ for MDA-MB-231 and BT549; 10^5^ for MCF7). The chemoattractant in the lower chamber was medium supplemented with 5% FBS (for MDA-MB-231 and MCF7 cells), or 1% FBS (for BT549 cells). After a certain time of migration (24 h for MCF7, 6 h for MDA-MB-231, 4 h for BT549), cells were fixed and stained with crystal violet. Migrated cells were quantified by counting five randomly chosen fields. The experiments were performed in duplicate wells and each experiment was performed at least three times as indicated.

### Trypan Blue Assay

MDA-MB-231 or BT549 cells (1×10^5^) were seeded in 12-well plates and after 16 h were treated with PK 11195 or Ro5-4864 and/or lonidamine or DMSO (vehicle) at the indicated concentrations. After 24 h, 0.4% solution of trypan blue in buffered isotonic salt solution was used to stain dead cells. The number of blue-staining cells and the number of total cells were quantified using a hemacytometer. Percentage of cell death was expressed as blue-staining cells/total cells ×100%.

### Statistical Analysis

For 3D morphogenesis assays, box plots were generated to represent the quantified data using either OriginLab software or web-based programs from http://www.physics.csbsju.edu/stats/t-test.html. For migration assays and trypan blue assays, bar graphs with mean±standard deviation were generated to represent the data. To compare the difference among experimental groups, student's *t*-tests were conducted and p<0.05 was considered as statistically significant.

## Results

### Stable Expression of TSPO in Human Mammary Epithelial MCF10A Cells

**Figure 1:**
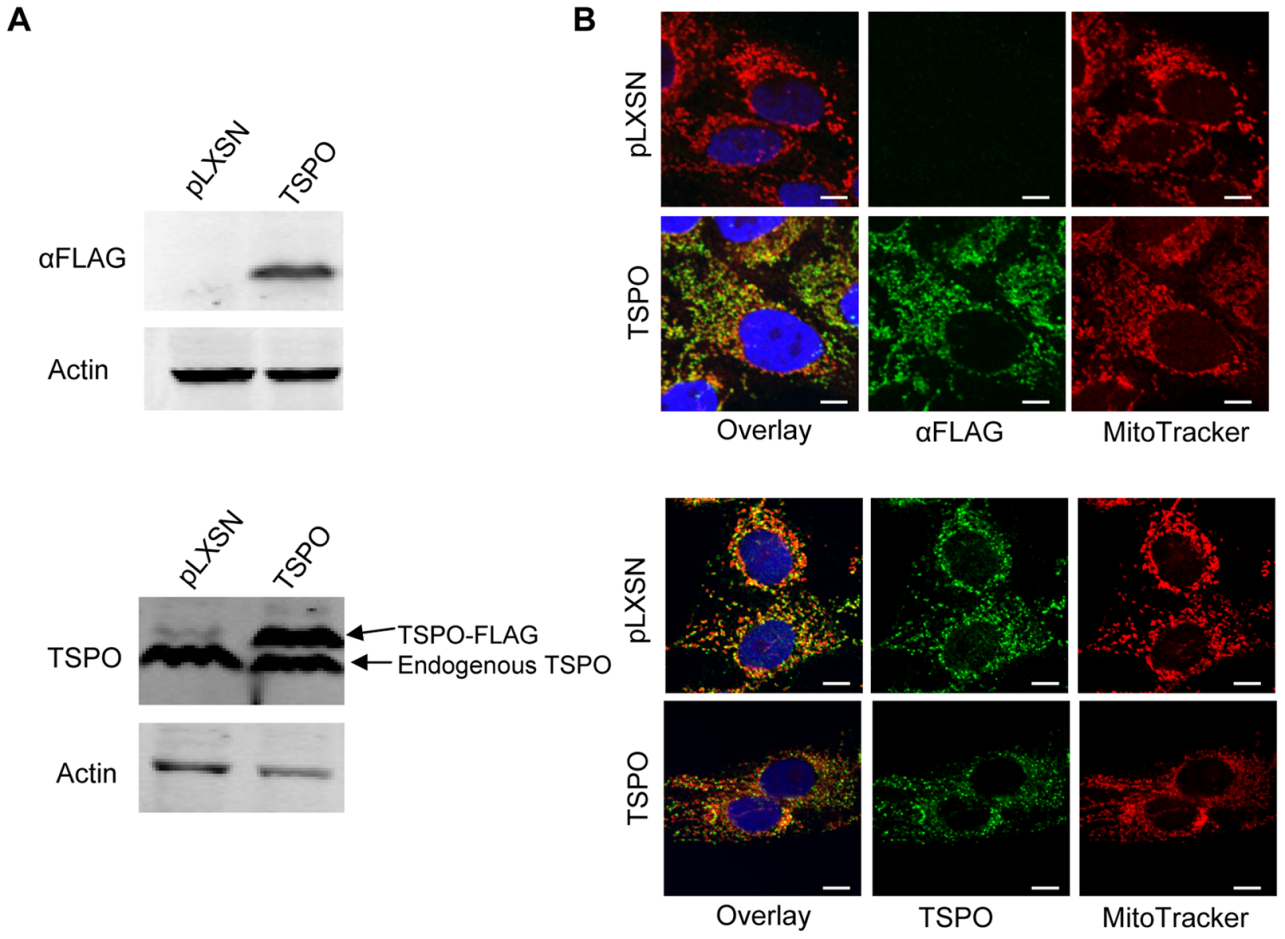
MCF10A cells stably overexpressing TSPO. Stable vector control (pLXSN) and TSPO-FLAG overexpressing cells (TSPO) were generated as described under *Materials and Methods*. **A**. Expression of TSPO was detected by immunoblotting using antibodies against FLAG tag (*top* panel) or TSPO (*bottom* panel), respectively, with actin as a loading control. **B**. Immunostaining of ectopically expressed and endogenous TSPO using antibodies against FLAG tag (*top* panel) or TSPO (*bottom* panel). MitoTracker Red was used to stain mitochondria and cells were imaged by confocal microscopy. Scale bar: 10 μm.

To investigate the role of TSPO in mammary morphogenesis, a stable pool of TSPO-overexpressing mammary epithelial cells, MCF10A-TSPO, was generated after retroviral transduction with a pLXSN construct encoding recombinant TSPO with a C-terminal FLAG epitope tag. A control MCF10A cell line pool expressing the empty vector pLXSN (MCF10A-pLXSN) was generated at the same time. The levels of ectopically expressed and endogenous TSPO in the stable MCF10A populations were evaluated by immunoblotting using an anti-FLAG (Fig. 1A, *top* panel) or anti-TSPO antibody (Fig. 1A, *bottom* panel). Ectopically expressed TSPO did not affect the endogenous level of TSPO. Based upon densitometry, total TSPO levels were 2.5 fold higher in the MCF10A-TSPO population compared to the control MCF10A-pLXSN population. The subcellular localization of endogenous and ectopically expressed TSPO in the population of MCF10A-TSPO cells showed similar distribution as judged by immunofluorescence staining with anti-FLAG (Fig. 1B, *top* panel) or anti-TSPO (Fig. 1B, *bottom* panel) antibody. FLAG-tagged TSPO was stably expressed in about 90% of the cells in the stable MCF10A population (data not shown). Confocal imaging revealed that both endogenous and ectopically expressed TSPO co-localized largely with MitoTracker Red, a mitochondrial marker, indicating that both endogenous and exogenous TSPO localize to the mitochondria.

### Elevation of TSPO Levels Increases Acinar Size and Promotes Proliferation during Mammary Epithelial Morphogenesis

**Figure 2:**
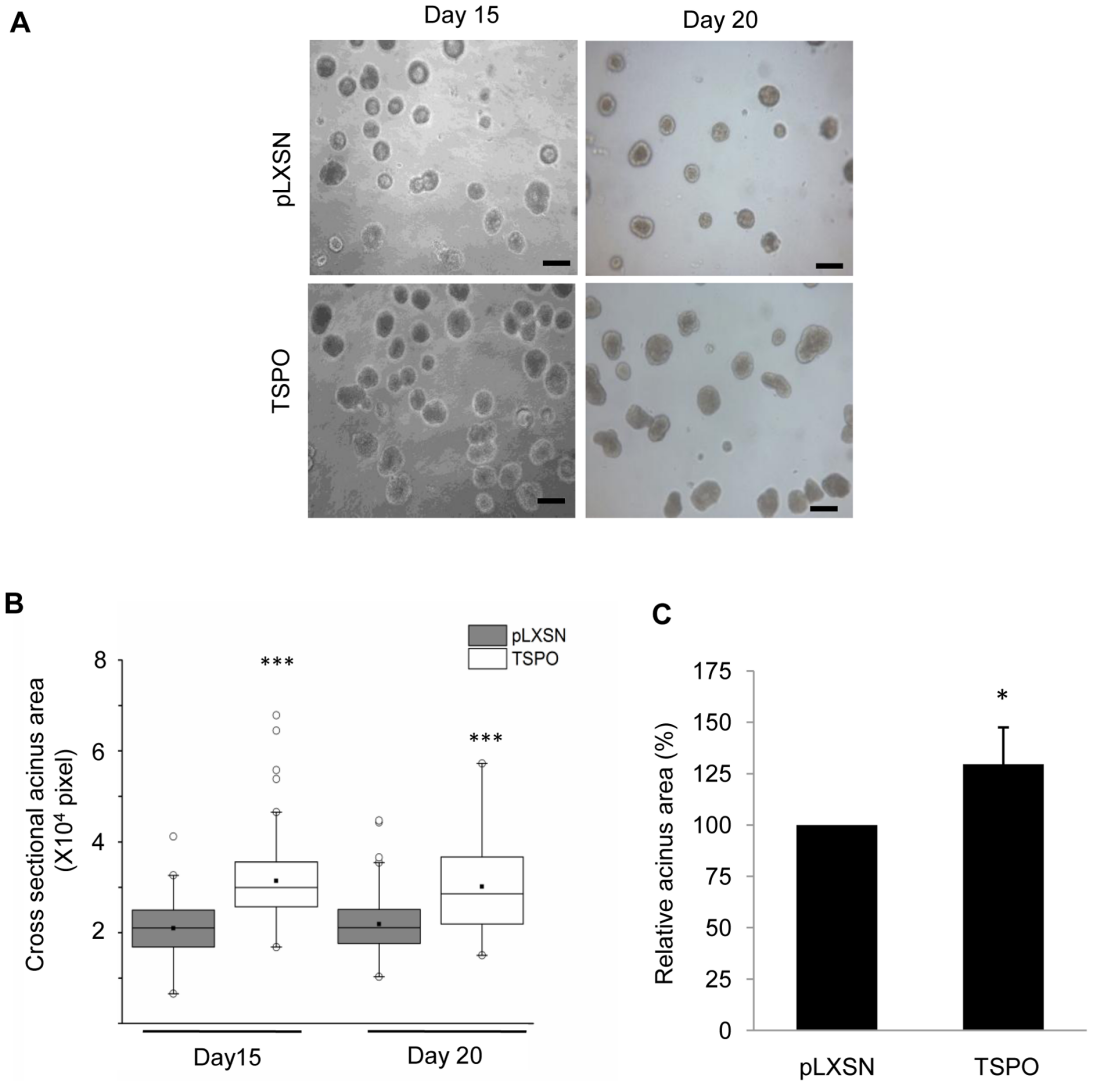
Overexpression of TSPO increases acini size. Control MCF10A-pLXSN and MCF10A-TSPO cells were seeded in Matrigel as described under *Materials and Methods*. **A**. Images were acquired on day 15 and day 20 of culture. Scale bar: 50 μm. **B**. Maximal cross-sectional area in pixels of individual acini was determined using ImageJ software, and plotted as a box plot. *Black line*, median value; *box*, interquartile range; *solid square*, mean; *open circles*, outliers. Data are from a representative set of three independent experiments in which ∼100 acini per condition were measured. P-value determined by Student's *t*-test. *** p<0.001 indicates a significant difference between TSPO and pLXSN control. **C**. The cross-sectional area of stable TSPO-overexpressing acini relative to the control acini ( = 100%). Each column represents results from 3 independent experiments in which a total of at least 300 acini per condition were measured. Error bar: SD. P-value was determined by Student's *t*-test. * p<0.05 indicates significant difference between TSPO and pLXSN control.

To evaluate the potential role(s) of TSPO in breast cancer development, the morphogenesis of MCF10A-TSPO and control MCF10A-pLXSN cells in 3D Matrigel culture was monitored over time. Images of cultures at day 15 and day 20 were acquired and used to evaluate the size of acini (Fig. 2A). Both control and TSPO-overexpressing acini reached their maximal size by day 15 (Fig. 2B). The elevation of TSPO expression resulted in a 30% increase on average in acinar cross-sectional area compared to the control vector-expressing acini (Fig. 2C). Quantitative analysis of maximal cross sections obtained by confocal imaging showed that the outer layer of MCF10A-TSPO acini had both a greater number of cells and a higher cell density than the control pLXSN acini (Supplementary Fig. S1). At earlier times (day 5 and day 8) TSPO-expressing structures were also modestly larger than pLXSN-control structures (Supplementary Fig. S2).

**Figure 3:**
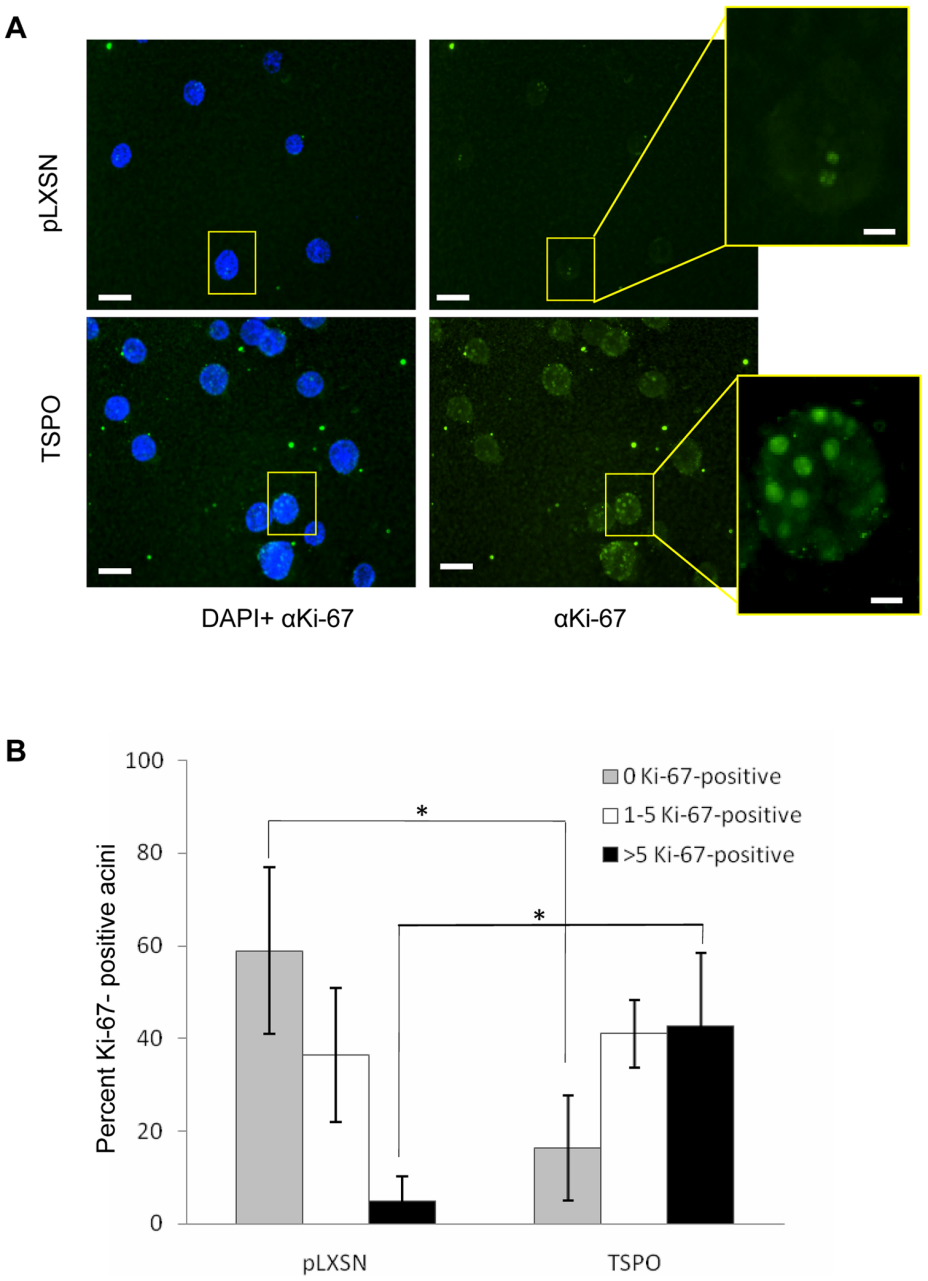
Overexpression of TSPO enhances proliferation during mammary epithelial morphogenesis. Control MCF10A-pLXSN cells and MCF10A-TSPO cells were seeded and cultured in Matrigel as described under *Materials and Methods*. **A**. On day 15, the cultures were fixed and stained with 4′,6′-diamidino-2-phenylindole (DAPI, *blue*) and anti-Ki-67 (*green*). Representative fluorescent images of control vector (pLXSN) and TSPO expressing acini are shown. Scale bar: 50 μm. Expanded image of a single acinus from pLXSN control or TSPO are shown. Scale bar: 10 μm. **B**. Cultures were scored for the number of acini containing 0, 1 to 5, or more than 5 Ki-67-positive cells based on at least 250 acini from each condition, combined from three independent experiments. Error bar: SD. P-value was determined by Student's *t*-test. * p<0.05 indicates a significant difference between TSPO and pLXSN control.

To investigate whether TSPO-induced proliferation contributes to the enlarged acinar phenotype, the number of proliferating cells within individual acini was quantified from immunofluorescence images acquired after staining with the proliferation marker Ki-67 at day 15, when cells of MCF10A acini are known to cease proliferation [26]. While no Ki-67 staining was observed in the majority of the control structures, most MCF10A-TSPO structures were Ki-67 positive (Fig. 3A), with at least one Ki-67 positive cell in 84% of MCF10A-TSPO acini, compared to 41% of control structures. More than 5 Ki-67 positive cells were observed in 43% of MCF10A-TSPO acini, compared to 5% of control MCF10A structures (Fig. 3B). In agreement with our findings at day 15, more Ki-67 positive cells were observed in MCF10A-TSPO acini at day 10 of mammary morphogenesis compared with control pLXSN structures (Supplementary Fig. S3). These data demonstrate that elevated expression of TSPO promotes proliferation during mammary epithelial morphogenesis, and that the enlarged acini observed upon TSPO overexpression is due, at least in part, to enhanced proliferation.

### Increased Expression of TSPO Results in Partially Filled Lumen during Morphogenesis

**Figure 4:**
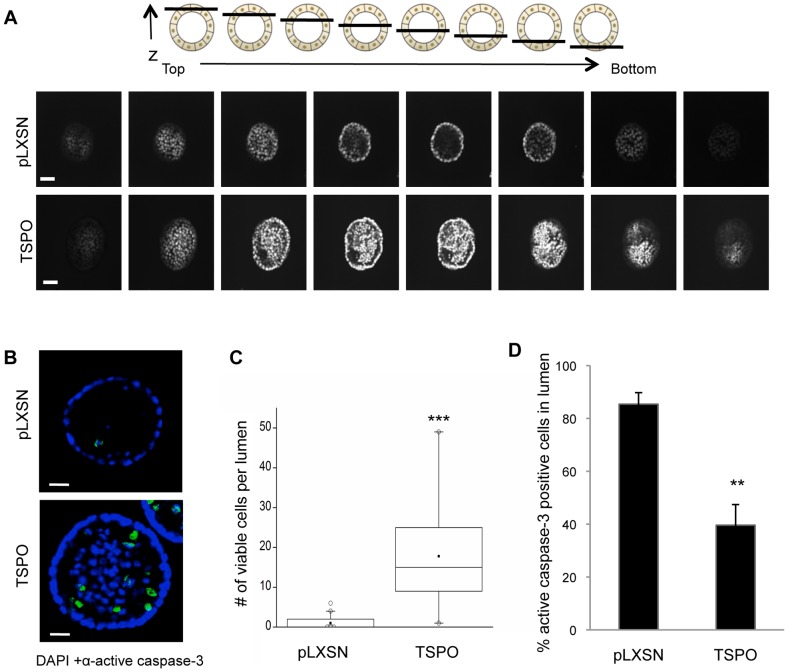
Overexpression of TSPO leads to partial filling of lumen during morphogenesis. Control MCF10A-pLXSN and MCF10A-TSPO cells were seeded and cultured in Matrigel. On day 20, cultures were fixed and stained with DAPI and anti-cleaved caspase-3 as described in *Materials and Methods*. **A**. Schematic diagram (*top* panel) of serial confocal cross sections of an acinus structure showing the relative position of the sections with respect to z-axis. Serial confocal cross-sections images of MCF10A-pLXSN and MCF10A-TSPO acini stained with DAPI (*bottom* panel). Scale bar: 20 μm. **B**. Representative images of cleaved caspase-3 staining (*green*) were acquired using confocal microscopy. Scale bar: 25 μm. **C**. The number of viable cells (active caspase-3 negative) per lumen was quantified, and plotted as a box plot. *Black line*, median value; *box*, interquartile range; *solid square*, mean; *open circles*, outliers. Data are from a representative set of three independent experiments in which ∼90 acini per condition were measured. **D**. The percent of active caspase-3 positive cells in the lumen was quantified based on at least 250 acini combined from three independent experiments. Error bar: SD. P-value was determined by Student's *t*-test. ** p<0.01, *** p<0.001 indicate significant differences between TSPO and pLXSN control.

To investigate the impact of increased TSPO levels on lumen formation during morphogenesis, nuclei of control and TSPO-overexpressing acini were stained with DAPI at day 20, and serial confocal cross sections were examined for the presence of DAPI-stained cells within the lumen. While MCF10A-control acini were hollow at day 20 as expected, the TSPO-overexpressing acini were partially filled (Fig. 4A).

Both enhanced proliferation and inhibition of apoptosis can result in the accumulation of cells within the lumen [26]. We speculated that overexpression of TSPO may prevent apoptosis of luminal cells, resulting in incomplete clearance of cells from the lumen, a process that has been shown to involve caspase-3 [26]. To assess whether increased expression of TSPO inhibits luminal apoptosis, the presence of cleaved, activated caspase-3 in luminal cells was detected by immunofluorescence. Activated caspase-3 was detected in the lumen of both control and TSPO-expressing acini during morphogenesis at day 10, day 15 (data not shown) and day 20 (Fig. 4B), suggesting TSPO overexpression does not completely suppress apoptosis. At the late stage of morphogenesis (day 20), only rarely were cells detected in the lumen of control acini, whereas large numbers of both viable (activated caspase-3 negative) and apoptotic (activated caspase-3 positive) cells were present in the lumen of the TSPO-overexpressing acini (Fig. 4B). Quantification showed that TSPO-overexpressing acini retained on average 10-fold more viable cells within their lumen compared to control acini (Fig. 4C). Of the luminal cells in control acini nearly 90% were active caspase-3 positive, whereas in TSPO-overexpressing acini only 40% of the luminal cells expressed active caspase-3 (Fig. 4D). These data, taken together, suggest that increasing TSPO levels partially suppresses luminal apoptosis.

### TSPO Promotes Breast Cancer Cell Migration

**Figure 5:**
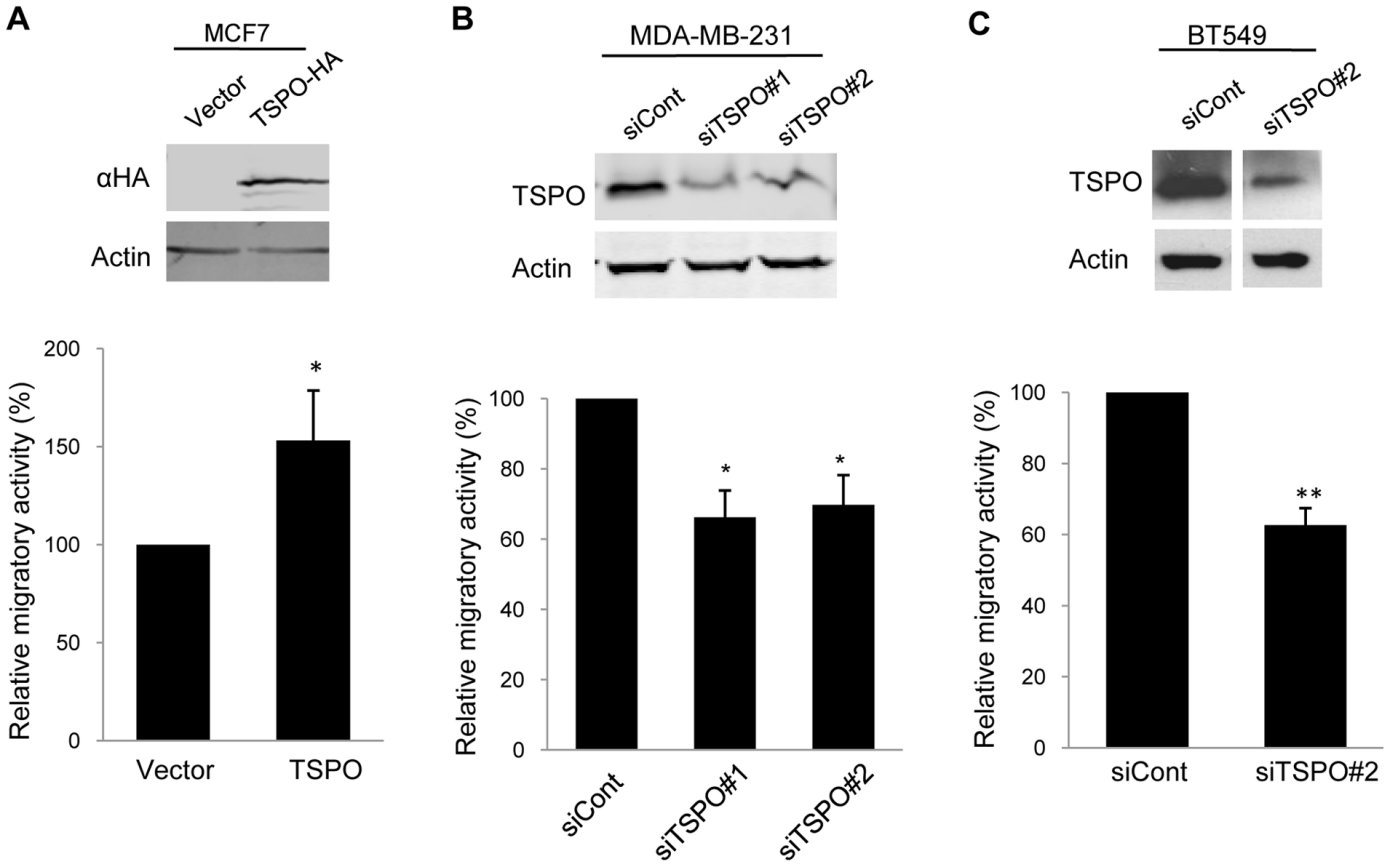
TSPO promotes breast cancer cell migration. **A.** Control or TSPO expression vectors were transiently expressed in MCF7 cells. HA-tagged TSPO was detected by immunoblotting using an antibody against the HA tag, with actin as a loading control (*top* panel). The control and HA-TSPO-expressing MCF7 cells were allowed to migrate toward 10% FBS for 24 h in a transwell assay as described under *Materials and Methods.* Duplicate wells were used for each of three independent experiments. The results are expressed relative to the migration of control cells ( = 100%). Column (*bottom* panel): Mean of three experiments. Error bar: SD. P value was determined by Student's *t*-test. * p<0.05.indicates a significant difference between control and TSPO-overexpressing MCF7 cells. Control siRNA and siRNA sequences against TSPO (siTSPO #1 or siTSPO #2) were used to transfect MDA-MB-231 cells (**B**) or BT549 (**C**). The extent of silencing was determined by immunoblotting with antibodies against TSPO**,** with actin used as a loading control (*top* panel). Control and TSPO-depleted cells were then subjected to transwell assays as described under *Materials and Methods* Duplicate wells were used for each of three independent experiments. The results are expressed relative to the migration of control cells ( = 100%). Column (*bottom* panel): Mean of three experiments. Error bar: SD. P-value was determined by Student's *t*-test. * p<0.05 and ** p<0.01 indicate significant differences between control and TSPO-depleted cells.

In addition to enhanced proliferation and resistance to apoptosis, breast cancer cells must acquire migratory ability for invasion and metastasis [6]. To examine the contribution of TSPO to breast cancer cell migration, we modulated the levels of TSPO by transient overexpression or silencing and performed transwell migration assays. Migrated cells were stained with crystal violet and quantified as described *Materials and Methods* (Supplementary Fig. S4). TSPO was overexpressed in a poorly migratory ER-positive breast cancer cell line, MCF7, by transient transfection and migration was assessed in a transwell assay. Ectopic expression of HA-tagged TSPO increased migration of MCF7 cells by 1.5 fold (Fig. 5A and Supplementary Fig. S4A). In complementary experiments, we examined whether silencing of TSPO could suppress migration of highly invasive ER-negative breast cancer cells. Two different siRNAs, siTSPO #1 and siTSPO #2, were used to silence the *TSPO* gene in MDA-MB-231 cells. Transient transfection of each siRNA decreased TSPO expression by 70%, as determined by quantitative immunoblotting (Fig. 5B, *top* panel). Using a commercially available TSPO antibody, we observed reduction of the immunoreactive 18 kDa band upon TSPO silencing, but did not observe changes in other immunoreactive bands (Supplementary Fig. S5). Transwell migration of the TSPO-depleted MDA-MB-231 cells decreased by about 35% compared with control siRNA-transfected MDA-MB-231 cells (Fig. 5B, *bottom* panel and Supplementary Fig. S4B). Similar results were obtained upon silencing of TSPO in another ER-negative breast cancer cell line, BT549, which resulted in 40% decrease in migration in a transwell assay (Fig. 5C and Supplementary Fig. S4C).

### Combination of TSPO Ligands with Lonidamine Potentiates Apoptosis in Breast Cancer Cells

**Figure 6:**
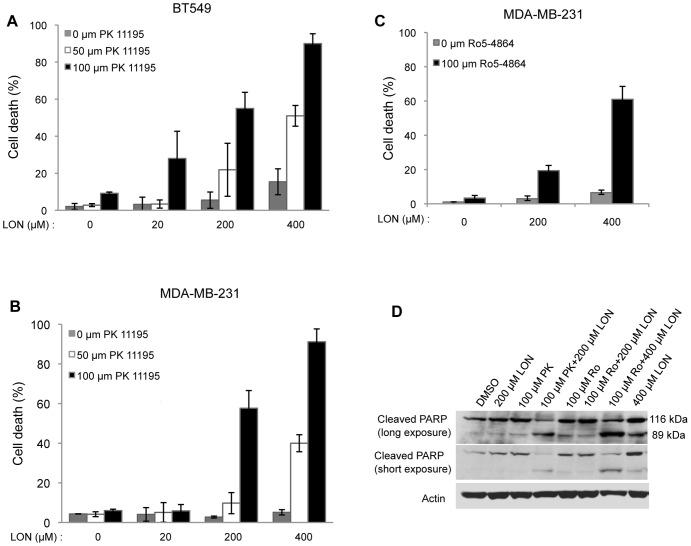
Combination of TSPO ligands and lonidamine potentiates apoptosis in breast cancer cells. BT549 (**A**) or MDA-MB-231 (**B**) cells were treated with vehicle (DMSO) or different concentrations of PK 11195 combined with different concentrations of lonidamine (LON) as indicated for 24 h. **C.** MDA-MB-231 cells were treated with DMSO or Ro5-4864 combined with different concentrations of LON, as indicated, for 24 h. Cell death was determined using trypan blue exclusion assays as described in *Materials and Methods*. Column: Mean of three independent experiments. Error bar: SD. **D**. MDA-MB-231 cells were treated with DMSO, PK 11195 or Ro5-4864 alone or in combination with LON at the indicated concentrations for 24 h and cellular lysates were generated. Immunoblotting of cellular lysates was performed using antibodies against PARP. Actin was used as loading control. PK: PK 11195; Ro: Ro5-4864.

Our data demonstrate important roles of the mitochondrial protein TSPO in driving phenotypes associated with breast cancer malignancy, suggesting it could be a potential therapeutic target. The isoquinoline carboxamide TSPO ligand, PK 11195 sensitizes hepatocellular carcinoma cells to cytotoxic chemotherapy agents [5], [21]. Ro5-4864, a benzodiazepine TSPO ligand, enhances apoptosis induced by chemotherapeutic agents in Jurkat cells [22]. Here, we tested the effect of combining PK 11195 or Ro5-4864 with lonidamine, another drug that targets the mitochondria, on viability using two ER-negative human breast cancer cell lines, MDA-MB-231 and BT549. Cells were treated with the indicated concentrations of PK 11195 and lonidamine for 24 h. The percentage of cell death was determined using trypan blue exclusion assays. As shown in Fig. 6 A and B, lonidamine alone (*grey bars*) or PK 11195 alone, over a range of concentrations, failed to induce cell death, whereas combining both PK 11195 and lonidamine dramatically induced cell death. For instance, in MDA-MB-231 cells, either 50 µM PK 11195 or 400 µM lonidamine alone did not induce cell death compared to vehicle control, whereas a combination of 50 µM PK 11195 and 400 µM lonidamine resulted in 40% cell death. In both MDA-MB-231 and BT549 cells, combining 100 µM PK 11195, with lonidamine resulted in 55% cell death at 200 µM and 90% cell death at 400 µM lonidamine. Like PK 11195, treatment with 100 µM Ro5-4864 alone had no effect on viability of MDA-MB-231 cells, whereas the combination of 100 µM Ro5-4864 and 400 µM lonidamine resulted in 60% cell death (Fig. 6C). To determine whether the cytoxicity induced by the combination of TSPO ligands with lonidamine is due to apoptosis, cleavage of poly (ADP-ribose) polymerase (PARP) was monitored (Fig. 6D). During apoptosis, the 116 kDa full length PARP is cleaved by caspase-3 to generate an 89 kDa fragment which is a well-validated apoptotic marker [31]. Upon treatment with a TSPO ligand, PK 11195 or Ro5-4864, or lonidamine alone, full length intact PARP (116 kDa) was observed. However, combined treatment with 100 µM PK 11195 and 200 µM lonidamine, or 100 µM Ro5-4864 and 400 µM lonidamine, resulted in PARP cleavage, as evidenced by the presence of the 89 kDa PARP fragment. These data demonstrate that combining a TSPO ligand and lonidamine efficiently induces apoptosis of ER-negative breast cancer cells. To determine whether mammary acini overexpressing TSPO would show similar sensitivity to TSPO ligands and lonidamine, the acini were treated for 48 h with PK 11195 alone or with PK 11195 and lonidamine. As shown in Supplementary Fig. S6, treatment with PK 11195 alone did not increase apoptosis as judged by active caspase-3 staining, but the combination of PK 11195 and lonidamine dramatically increased apoptosis of cells both in the lumen and in the outer layer.

## Discussion

Understanding the roles of the mitochondrial proteins in breast cancer, particularly those with differential expression in the diseased state, is critical for developing treatment strategies [5], [7]. Increased levels of TSPO correlate with increasing invasiveness of breast cancer tissues and breast cancer cells [13], [15], [16]. We hypothesized that increasing TSPO levels at each stage of breast cancer may confer distinct malignant properties to mammary epithelial and breast cancer cells that promote breast cancer progression. Thus, we used non-tumorigenic, immortalized mammary epithelial MCF10A cells, non-invasive ER-positive MCF7 breast cancer cells and invasive ER-negative MDA-MB-231 and BT549 cells as our cell line models for different stages of breast cancer.

Hyperproliferation and filling of the luminal space are key features of atypical hyperplasia and DCIS [24], [25]. Oncogene-induced breast cancer phenotypes have been modeled using 3D Matrigel culture of MCF10A cells [24]. During morphogenesis of MCF10A acini, some of the proliferative outer layer cells are deposited into the lumen [26]. To form and maintain a hollow lumen, luminal cells undergo apoptosis, presumably due to the lack of matrix-derived survival signals [23], [24]. Inappropriate proliferation of the outer layer cells along with decreased apoptosis of the luminal cells can lead to formation of aberrant acini with filled lumen [26]. Overexpression of cyclin D1 or HPV-E7 in MCF10A cells leads to formation of larger acini but the lumens remain hollow, consistent with the established role of these oncogenes in proliferation [26]. In these model systems, luminal filling is only observed upon coexpression with anti-apoptotic Bcl family members, supporting the notion that increased proliferation and resistance to apoptosis are both required for luminal filling. In contrast, elevation of TSPO levels is sufficient to produce enlarged acini with partially filled lumens, and in these respects more closely resembles the phenotypes induced by activated Akt [27], mixed-lineage kinase 3 [32], ErbB2/HER2 [26], [28] or B-Raf^V600E^ [33].

Our data are consistent with the idea that elevation of TSPO promotes both proliferation of outer layer cells and survival of cells within the lumen. At day 15 expression of TSPO dramatically increases proliferation compared to control acini (Fig. 3). By day 20, however, only rarely are proliferative cells observed in TSPO-expressing acini (data not shown), yet large numbers of viable cells remain in the lumen (Fig. 4). We speculate that the increased levels of TSPO in these viable luminal cells afford at least partial protection from apoptosis. Although the role of TSPO in the regulation of apoptosis is not completely defined, a body of evidence supports an anti-apoptotic function of TSPO. TSPO physically associates with the mitochondrial permeability transition pore (PTP) complex and thus increased TSPO levels may increase resistance to mitochondrial membrane permeability and subsequent cell death [12]. In chronic lymphocytic leukemia (CLL) cells, PK 11195 has been shown to induce apoptosis by mitochondrial membrane depolarization along with cytochrome c release [34].

Increased migratory ability is critical in the transition from localized to invasive breast cancer. Our data demonstrate that increasing expression of TSPO promotes migration of poorly migratory MCF-7 cells, whereas, silencing of TSPO decreased migration of invasive MDA-MB-231 cells. These data provide evidence for the idea that increased TSPO levels contribute to acquisition of an invasive phenotype in breast cancer cells. These findings are consistent with studies in rat glioma cells [35], suggesting that TSPO might regulate migration and invasion in a wide range of tumor types. In 3D Matrigel mammary, acini derived from TSPO-overexpressing nontumorigenic MCF10A mammary epithelial cells do not show an invasive phenotype, suggesting that, at least in this context, TSPO alone is not sufficient to induce invasion. While the underlying mechanism whereby TSPO influences cell migration is unknown, it is conceivable that TSPO affects cellular energy supply necessary for migration by perturbing the function of adenine nucleotide translocase (ANT), which is also found in the PTP complex. ANT is responsible for ATP and ADP exchange between mitochondria and cytosol and plays an essential role in cellular energy metabolism [36], [37].

Lonidamine is a mitochondria-targeting agent that disrupts aerobic glycolysis [5], likely through inhibition of the mitochondrial bound hexokinase 2 [38], [39], although ANT has also been suggested as a target [40], [41]. Our data show that combined treatment of TSPO ligands, PK 11195 or Ro5-4864, with lonidamine greatly reduces viability of MDA-MB-231 cells, compared with either single agent, consistent with findings in myeloid cells [41]. Our findings that PK 11195 and Ro5-4864, which represent different classes of TSPO ligands, give similar results when combined with lonidamine imply that TSPO is a potential drug target and suggest that dual targeting of mitochondria could be a useful therapeutic approach for treating ER-negative breast cancer. While no toxicity of PK 11195 has been observed in small human trials [42], a phase II trial of lonidamine in metastatic breast cancer patients was halted due to toxicity [43]. Our data suggest that lower doses of lonidamine might be therapeutically efficacious when combined with TSPO ligands.

Clinical observations show incremental increases in TSPO levels from normal breast tissues through advancing stages of breast cancer. Using a 3D mammary epithelial morphogenesis model, our studies suggest a novel role for TSPO in luminal cell survival, a key feature of DCIS. Increased TSPO levels promote proliferation in the 3D mammary acini, consistent with findings in 2D culture of breast cancer cell lines [16], [19]. Our studies further show that altering TSPO levels impacts breast cancer cell migration, a necessary property for invasion and metastasis. Our results suggest that PK 11195, Ro5-4864 or other TSPO ligands, might be useful in developing combination therapies for breast cancer treatment.

## Supporting Information

Figure S1 **Stable TSPO overexpression increases the number and density of cells within the outer layer of acini.** MCF10A-pLXSN and MCF10A-TSPO cells were seeded in Matrigel as described under *Materials and Methods*. Confocal images were acquired on day 15. The number of outer layer cells within, and the circumference of the maximal acinar cross section were quantified from confocal images using ImageJ software. The results are expressed relative to number of cells in the outer layer in the circumference of MCF10A-pLXSN acini ( = 100%) (*left* panel). Cell density within the outer layer was calculated as cell number of outer layer/circumference in arbitrary units. The results are expressed relative to outer layer cell density of MCF10A-pLXSN acini ( = 100%) (*right* panel). Results are based on 300 acinar structures for each condition, combined from three independent experiments. P-value was determined by Student's *t*-test. * p<0.05 indicates significant differences between TSPO-expressing acini and pLXSN control acini.
(TIFF)

Figure S2 **Overexpression of TSPO increases acini size at early stages of mammary morphogenesis.** MCF10A-pLXSN (control) and MCF10A-TSPO cells were seeded in Matrigel as described under *Materials and Methods*. Confocal images were acquired on the indicated days. Cross-sectional area in pixels of individual acini from day 5 and day 8 was determined using ImageJ software, and plotted as a box plot (*left panel*). *Black line*, median value; *box*, interquartile range; *solid square*, mean; *open circles*, outliers. Data are combined from ∼70 acini per condition. P-value was determined by Student's *t*-test. *** p<0.001 indicates a significant difference between TSPO-expressing and control (pLXSN) acini. Representative confocal images of control (pLXSN) and TSPO-expressing acini at day 8 are shown (*right panel*).
(TIF)

Figure S3 **Overexpression of TSPO increases proliferation at early stage of mammary morphogenesis.** MCF10A-pLXSN (control) and MCF10A-TSPO cells were seeded in Matrigel as described under *Materials and Methods*. At day 10, cultures of acini were fixed and stained with DAPI (*blue*) and anti-Ki-67 (*green*). Confocal images were acquired and representative images of control vector (pLXSN) and TSPO-expressing acini are shown (*left panel*). Scale bar: 20 μm. The number of Ki67-positive cells were quantified from at least 70 acini from each condition, and plotted as a box plot (*right panel*). *Black line*, median value; *box*, interquartile range; *open circles*, outliers. P-value was determined by Student's *t*-test. *** p<0.001 indicates a significant difference between TSPO and pLXSN control.
(TIF)

Figure S4 **Images of crystal violet-stained cells from transwell migration assays.** Transwell migration was performed as described in *Materials and Methods*. Migrated cells were stained with crystal violet at the times indicated in Fig. 5. Quantification from five randomly chosen fields is shown in Fig. 5. Images of one representative field from each condition are shown here. **A**. Control vector or TSPO-overexpressing MCF7 cells. **B**. Control or TSPO-depleted MDA-MB-231 cells. **C**. Control or TSPO-depleted BT549 cells
(TIF)

Figure S5 **Full immunoblot of cellular lysates from MDA-MB-231 cells after TSPO silencing.** The full TSPO immunoblot from Fig. 5 is shown. The 18 kDa TSPO band, as well as molecular weight markers, are indicated. The actin blot from Fig. 5 is shown as a loading control. See Fig. 5 and *Material and Methods* for details. Control siRNA and siRNA against TSPO (siTSPO #1 or siTSPO #2) were used to transfect MDA-MB-231 cells as described under *Materials and methods*. After 24 h, cellular lysates were harvested and subjected to immunoblotting assays using an anti-TSPO antibody, with actin as a loading control.
(TIF)

Figure S6 **Combination of PK 11195 and lonidamine increases apoptosis during mammary morphogenesis.** MCF10A-TSPO cells were seeded in Matrigel as described under *Materials and Methods*. At day 13, cultures were treated with vehicle (DMSO), or 100 μM PK 11195 or a combination of 100 μM PK 11195 and 200 μM lonidamine. After 48 h treatment, the cultures were fixed and stained with DAPI (*blue*) and anti-active caspase-3 (*green*). Representative fluorescence images are shown. Scale bar: 20 μm.
(TIF)
