# Supplementary figures and images for: The 18-kDa Translocator Protein (TSPO) Disrupts Mammary Epithelial Morphogenesis and Promotes Breast Cancer Cell Migration
Source: PLoS One. 2013 Aug 14;8(8):e71258. doi: 10.1371/journal.pone.0071258 (PMC3743866; doi:10.1371/journal.pone.0071258)

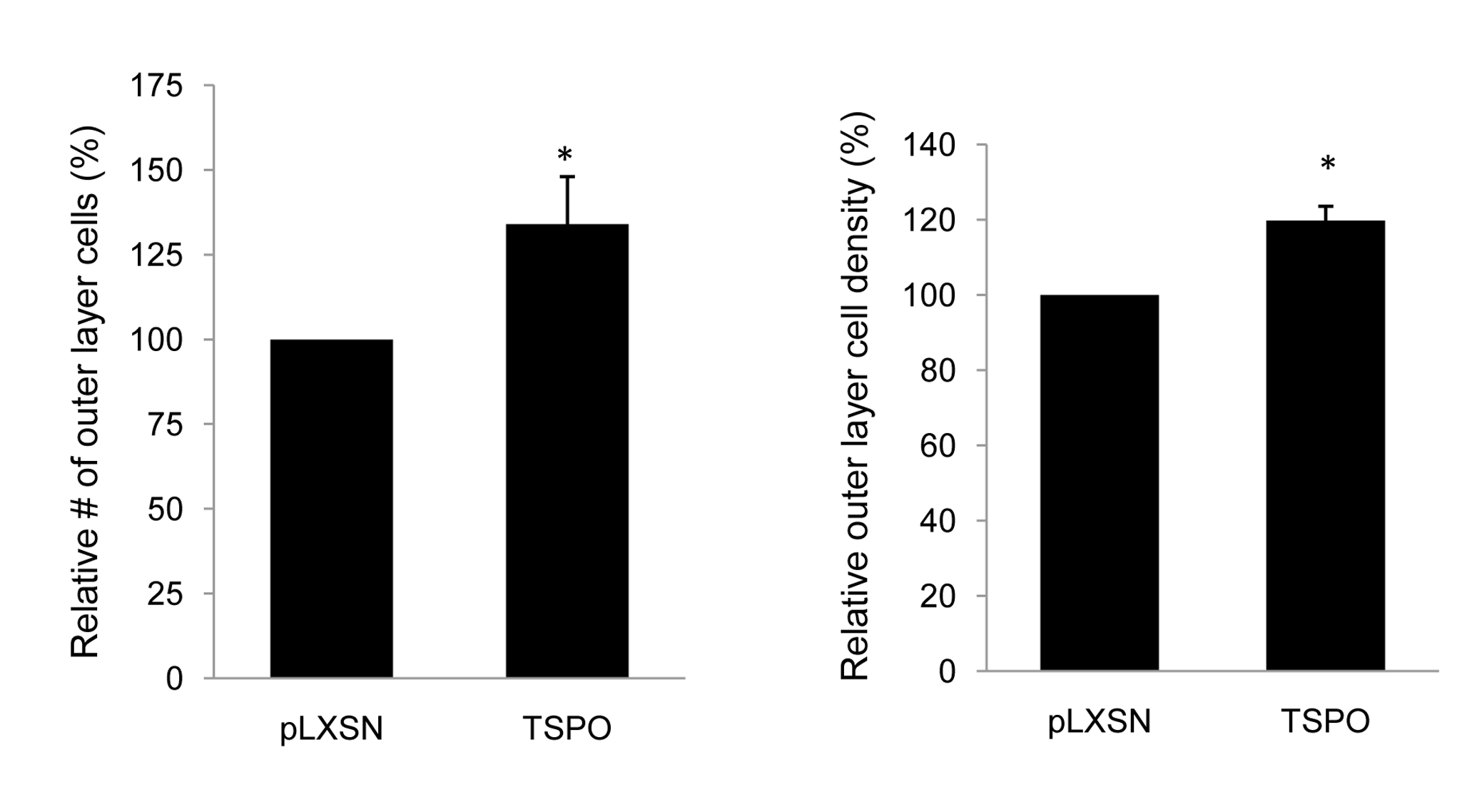

Supplement: Figure S1 — Stable TSPO overexpression increases the number and density of cells within the outer layer of acini. MCF10A-pLXSN and MCF10A-TSPO cells were seeded in Matrigel as described under Materials and Methods. Confocal images were acquired on day 15. The number of outer layer cells within, and the circumference of the maximal acinar cross section were quantified from confocal images using ImageJ software. The results are expressed relative to number of cells in the outer layer in the circumference of MCF10A-pLXSN acini ( = 100%) (left panel). Cell density within the outer layer was calculated as cell number of outer layer/circumference in arbitrary units. The results are expressed relative to outer layer cell density of MCF10A-pLXSN acini ( = 100%) (right panel). Results are based on 300 acinar structures for each condition, combined from three independent experiments. P-value was determined by Student's t-test. * p<0.05 indicates significant differences between TSPO-expressing acini and pLXSN control acini. (TIFF) [file pone.0071258.s001.tiff]

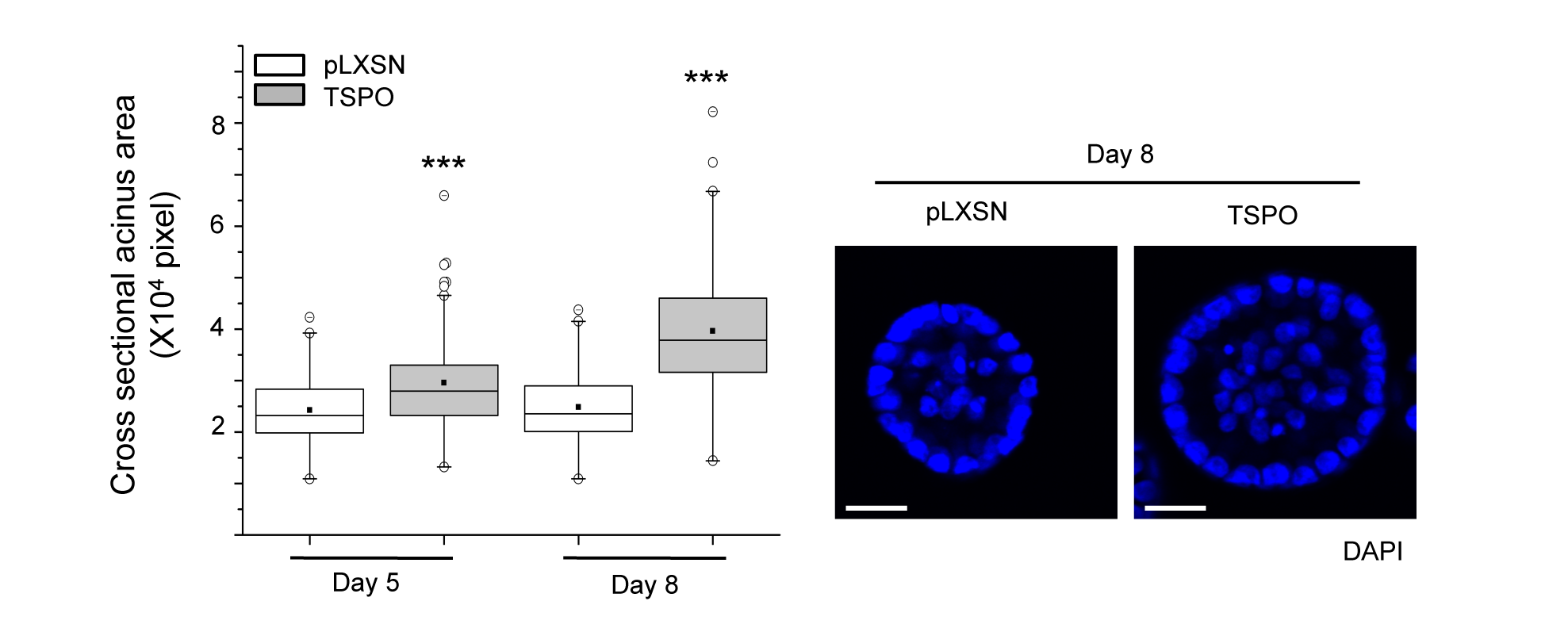

Supplement: Figure S2 — Overexpression of TSPO increases acini size at early stages of mammary morphogenesis. MCF10A-pLXSN (control) and MCF10A-TSPO cells were seeded in Matrigel as described under Materials and Methods. Confocal images were acquired on the indicated days. Cross-sectional area in pixels of individual acini from day 5 and day 8 was determined using ImageJ software, and plotted as a box plot (left panel). Black line, median value; box, interquartile range; solid square, mean; open circles, outliers. Data are combined from ∼70 acini per condition. P-value was determined by Student's t-test. *** p<0.001 indicates a significant difference between TSPO-expressing and control (pLXSN) acini. Representative confocal images of control (pLXSN) and TSPO-expressing acini at day 8 are shown (right panel). (TIF) [file pone.0071258.s002.tif]

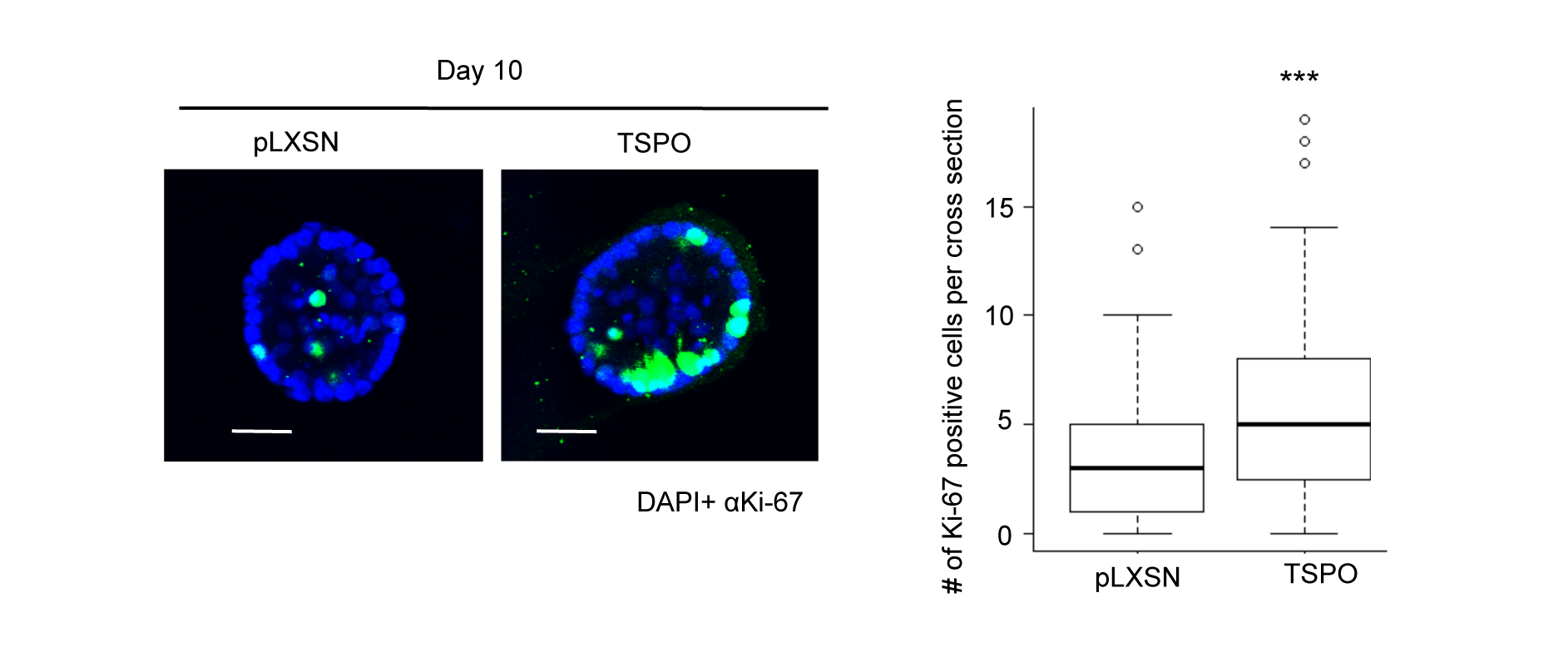

Supplement: Figure S3 — Overexpression of TSPO increases proliferation at early stage of mammary morphogenesis. MCF10A-pLXSN (control) and MCF10A-TSPO cells were seeded in Matrigel as described under Materials and Methods. At day 10, cultures of acini were fixed and stained with DAPI (blue) and anti-Ki-67 (green). Confocal images were acquired and representative images of control vector (pLXSN) and TSPO-expressing acini are shown (left panel). Scale bar: 20 μm. The number of Ki67-positive cells were quantified from at least 70 acini from each condition, and plotted as a box plot (right panel). Black line, median value; box, interquartile range; open circles, outliers. P-value was determined by Student's t-test. *** p<0.001 indicates a significant difference between TSPO and pLXSN control. (TIF) [file pone.0071258.s003.tif]

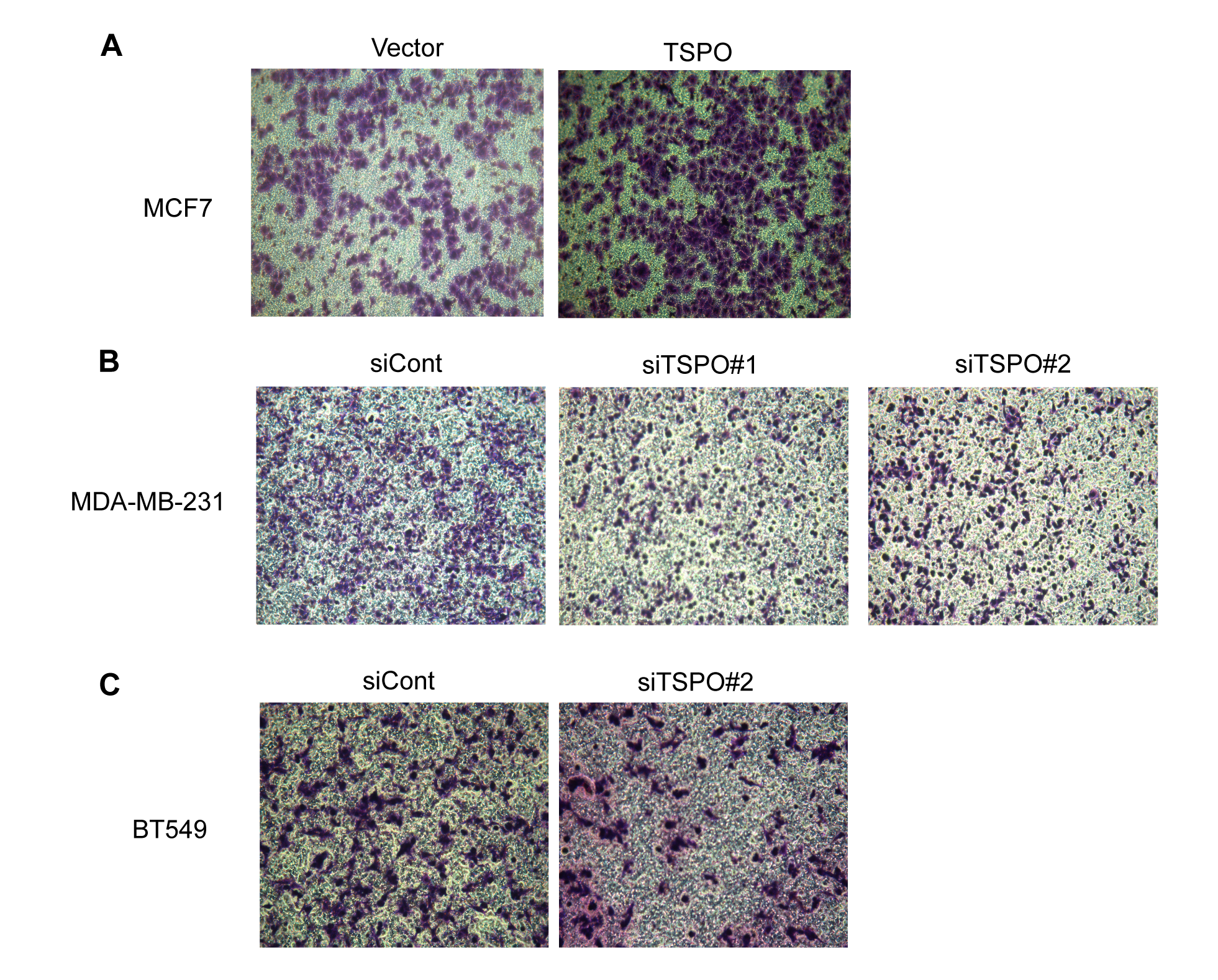

Supplement: Figure S4 — Images of crystal violet-stained cells from transwell migration assays. Transwell migration was performed as described in Materials and Methods. Migrated cells were stained with crystal violet at the times indicated in Fig. 5. Quantification from five randomly chosen fields is shown in Fig. 5. Images of one representative field from each condition are shown here. A. Control vector or TSPO-overexpressing MCF7 cells. B. Control or TSPO-depleted MDA-MB-231 cells. C. Control or TSPO-depleted BT549 cells (TIF) [file pone.0071258.s004.tif]

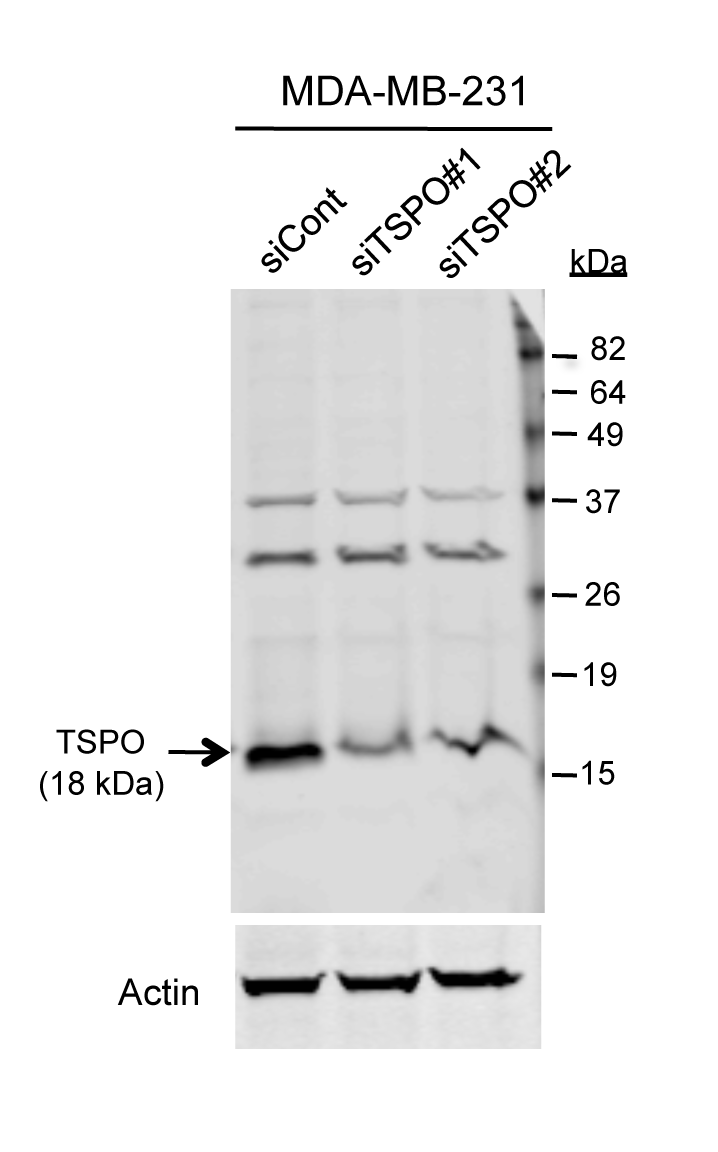

Supplement: Figure S5 — Full immunoblot of cellular lysates from MDA-MB-231 cells after TSPO silencing. The full TSPO immunoblot from Fig. 5 is shown. The 18 kDa TSPO band, as well as molecular weight markers, are indicated. The actin blot from Fig. 5 is shown as a loading control. See Fig. 5 and Material and Methods for details. Control siRNA and siRNA against TSPO (siTSPO #1 or siTSPO #2) were used to transfect MDA-MB-231 cells as described under Materials and methods. After 24 h, cellular lysates were harvested and subjected to immunoblotting assays using an anti-TSPO antibody, with actin as a loading control. (TIF) [file pone.0071258.s005.tif]

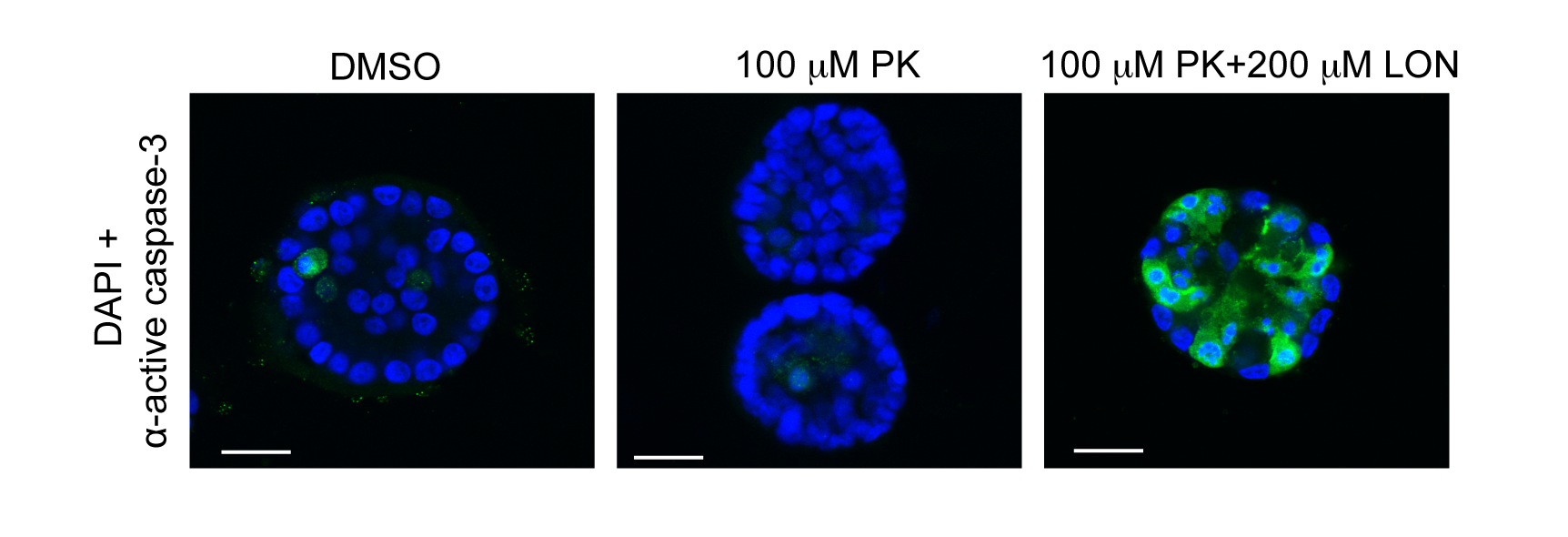

Supplement: Figure S6 — Combination of PK 11195 and lonidamine increases apoptosis during mammary morphogenesis. MCF10A-TSPO cells were seeded in Matrigel as described under Materials and Methods. At day 13, cultures were treated with vehicle (DMSO), or 100 μM PK 11195 or a combination of 100 μM PK 11195 and 200 μM lonidamine. After 48 h treatment, the cultures were fixed and stained with DAPI (blue) and anti-active caspase-3 (green). Representative fluorescence images are shown. Scale bar: 20 μm. (TIF) [file pone.0071258.s006.tif]
